# Autophagy and airway fibrosis: Is there a link?

**DOI:** 10.12688/f1000research.11236.2

**Published:** 2018-06-11

**Authors:** Anudeep Kota, Deepak A. Deshpande, Mehra Haghi, Brian Oliver, Pawan Sharma

**Affiliations:** 1 Faculty of Science, University of Technology Sydney, Sydney, NSW, 2007, Australia; 2 Woolcock Emphysema Centre, Woolcock Institute of Medical Research, The University of Sydney, Sydney, NSW, 2037, Australia; 3 Graduate School of Health, University of Technology Sydney, Sydney, NSW, 2007, Australia; 4 Centre for Translational Medicine, Thomas Jefferson University, Philadelphia, PA, 19107, USA

**Keywords:** asthma, COPD, airway remodeling, airway mesenchymal cells

## Abstract

In the past decade, an emerging process named “autophagy” has generated intense interest in many chronic lung diseases. Tissue remodeling and fibrosis is a common feature of many airway diseases, and current therapies do not prevent or reverse these structural changes. Autophagy has evolved as a conserved process for bulk degradation and recycling of cytoplasmic components to maintain basal cellular homeostasis and healthy organelle populations in the cell. Furthermore, autophagy serves as a cell survival mechanism and can also be induced by chemical and physical stress to the cell. Accumulating evidence demonstrates that autophagy plays an essential role in vital cellular processes, including tissue remodeling. This review will discuss some of the recent advancements made in understanding the role of this fundamental process in airway fibrosis with emphasis on airway remodeling, and how autophagy can be exploited as a target for airway remodeling in asthma and chronic obstructive pulmonary disease.

## Introduction

**Figure 1.**
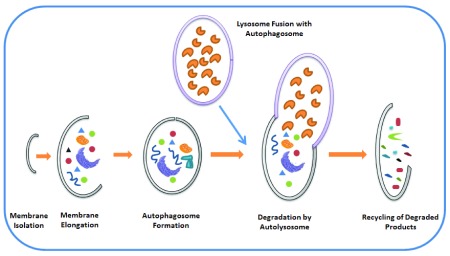
Pictorial representation of the autophagy pathway. The autophagy pathway proceeds through several phases, including initiation (formation of a preautophagosomal structure leading to an isolation membrane, or phagophore), vesicle elongation, autophagosome maturation and cargo sequestration, and autophagosome–lysosome fusion. In the final stage, autophagosomal contents are degraded by lysosomal acid hydrolases and the contents of the autolysosome are released for metabolic recycling.

Autophagy is an evolutionarily conserved pathway for the turnover of organelles and proteins by lysosomal-dependent processing ^1^. During autophagy, newly formed double-membrane structures, called autophagosomes, encapsulate cytoplasmic material, such as dysfunctional or damaged organelles or proteins. The autophagosomes then fuse with lysosomes, thus delivering the sequestered cargo for lysosomal-dependent degradation ^2^, as described in Figure 1. In the last decade, autophagy has emerged as a fundamental process involved in tissue and cellular homeostasis, and thus has been implicated in maintaining basal physiologic (healthy) and adaptive pathophysiologic responses (disease) ^2– 4^. There is increasing evidence to suggest that autophagy can impact the pathogenesis and/or progression of many human diseases ^2, 4, 5^, including neurodegenerative diseases ^6^, cancer ^7^, heart diseases ^8, 9^ and immune disorders (reviewed in 3, 10).

Fibrotic airway remodeling remains a key pathological feature correlating with a decline in lung function and disease progression in both asthma and chronic obstructive pulmonary disease (COPD) patients ^11– 13^. While there is no treatment for preventing development of airway remodeling, cell fate phenomena, such as autophagy, have been shown to play a role in asthma and COPD pathogenesis. Here, we review the progress in understanding how autophagy can contribute to airway fibrosis and the emerging strategies to target this process for therapeutic benefit.

## Evidence of autophagy in asthma

Asthma is a chronic inflammatory disease of the lungs, characterized by airway inflammation, airway hyperresponsiveness and tissue remodeling. It affects more than 300 million people worldwide and this number is estimated to escalate to 400 million by 2025 ^14^.

The role of autophagy in pulmonary diseases has gained attention in the past decade, but only very few studies have described the role of autophagy in asthma. One of the very first studies that described a direct role between asthma and autophagy revealed the association of the *ATG5* gene in the pathogenesis of asthma ^15^. This study found a genetic association in 1338 adult patients with asthma and the expression of the autophagy gene *ATG5*. *ATG5* encodes the ATG5 protein of 276 amino acids. During autophagy, the ATG5 protein interacts with ATG12 and ATG16 to form a ATG12-ATG5-ATG16 complex. This complex is associated with autophagosomal membrane elongation by interaction with ATG3, leading to ATG8-phosphatidyl ethanolamine formation ^16^. This association was further validated in another study with 312 asthmatic and 246 control children, which showed that genetic variants in *ATG5* are associated with pathogenesis of childhood asthma ^17, 18^. Furthermore, a study by Poon *et al.* revealed the role of *ATG5* in adult asthma, and also found an increased number of autophagosomes in fibroblast and epithelial cells from severe asthmatics when compared to healthy volunteers ^16^. Recent studies show that there is emerging evidence for the role of autophagy in both eosinophilic ^19^ and neutrophilic asthma ^20^, and convey its link to severe asthma and fibrotic tissue remodeling.

A recent study by Ban *et al.* investigated the role of autophagy in sputum granulocytes, peripheral blood cells and peripheral blood eosinophils of severe and non-severe asthmatics ^21^. They found increased autophagy in the immune cells from the severe asthmatics when compared to non-severe and healthy controls. This clearly indicates that induction of autophagy in immune cells is associated with severe asthma. By contrast, a study conducted by Akbari’s group reveals the induction of neutrophilic airway inflammation and hyperreactivity on deletion of CD11 cell specific *ATG5* mice. In addition, in this study augmented neutrophilic inflammation in Atg5(-/-) mice is IL-17A driven and glucocorticoid resistant ^22^.

In our own hands, we have found increased signatures of key autophagy genes in the lungs of asthmatic patients when compared with non-asthmatics, suggesting that basal autophagy is higher in asthma (unpublished data). Furthermore, we also found increased expression of autophagy proteins in the lung tissue obtained from chronic mouse model of HDM-induced asthma and this expression was found to correlate with pro-fibrotic signaling (Smad) and extracellular matrix protein (collagen) in the lung (unpublished data).

These data suggest that autophagy and airway fibrosis occur together with allergic insult, and act as a key driver for airway remodeling in allergic asthma. The current literature clearly indicates that the autophagy-phenomenon may be a crucial driver in the pathogenesis of asthma, particularly in severe forms of the disease, with an unknown underlying mechanism. The therapeutic modulation of autophagy with novel inhibitors may lead to the development of a new class of drugs for severe asthma.

## Evidence of autophagy in COPD

COPD is a progressive lung disease characterized by accelerated decline in lung function over time. Its most common pathological feature includes emphysema and chronic bronchitis. Airway obstruction in COPD in associated with formation of peribronchial fibrosis, increased wall thickness and excess mucus secretion, especially in the smaller airways ^23^. Exposure to cigarette smoke is one major cause of COPD; however only 25% of smokers develop COPD, which suggests the existence of numerous other factors contributing to COPD (such as genetic predisposition and oxidative stress) ^24, 25^. The role of autophagy in COPD seems to be more complex than anticipated, as some studies showed its impairment ^26– 28^, while others suggest it facilitates disease pathogenesis ^29– 32^. More recently, the role of selective autophagy (such as mitophagy, ciliophagy and xenophagy) in COPD pathology has been proposed ^32^.

The very first demonstration of autophagy in COPD was shown by Chen *et al.*, where authors found increased autophagy markers in the lungs of COPD patients ^29^. The authors also found a similar expression of elevated autophagy markers at various stages of the disease. This suggests that altered autophagy could be a key regulator in the pathogenesis and progression of COPD. The same study also showed that the autophagy marker LC3II/LC3I was significantly increased in lungs from patients with α-1 anti-trypsin deficiency when compared with non-COPD donors ^29^. In addition, many other studies have shown the increased expression of autophagy markers both *in vitro* and *in vivo* when exposed to cigarette smoke extract ^17, 29, 30, 33^, which explains increased loss of alveolar epithelial cells as seen in emphysema.

Moreover, to investigate the role of autophagy in chronic bronchitis, Lam and colleagues demonstrated that induction of autophagy leads to shortening of cilia in mouse tracheal epithelial cells exposed to cigarette smoke ^31^. They further found that autophagy gene deficient mice (Becn1 ^+/-^ or Map1lc3B ^-/-^) were resistant to the shortening of cilia in tracheal epithelial cells when exposed to cigarette smoke, demonstrating a direct role of autophagy in this process ^31^. Recent studies have demonstrated that selective autophagy (namely mitophagy) plays an important role in regulating mitochondrial function, which in turn has a crucial role in COPD pathogenesis ^34^. However, the specific role of mitophagy in tissue injury mediated by cigarette smoke remains obscure and requires further study ^32^.

Overall, autophagy plays a key role in COPD pathogenesis, especially in the development of emphysema, but the underlying mechanisms by which it promotes emphysema and bronchitis in COPD is not clear.

## Autophagy and fibrotic airway remodeling

The pathogenesis of COPD and asthma is typified by structural changes in the lung, collectively known as airway remodeling, which is characterized by basement membrane fibrosis, epithelial goblet cell hyperplasia, deposition of extracellular matrix proteins and smooth muscle hypertrophy ^35, 36^. Current therapies provide very limited benefit on airway remodeling ^12, 37– 41^, thus identifying new drug targets that can prevent or reduce airway remodeling in asthma and COPD is vital to reverse structural changes that determines the underlying cause of the disease ^35, 42^. TGFβ1 is a well-known regulator of inflammation and fibrotic remodeling in COPD and asthma, and it is upregulated in both diseases ^11^. TGFβ1 is the most abundant isoform of the TGF-β family and is secreted by most immune cells, including airway epithelial, smooth muscle, fibroblast and endothelial cells. TGFβ1 acts through the Smad dependent pathway and independent pathways, like mitogen activated protein kinases (MAPKs) namely p38 MAPK and c-Jun-N-terminal kinase, leading to the accumulation of extracellular matrix (ECM).

Autophagy-mediated TGFβ1-induced fibrosis plays a key role in the pathogenesis of heart and kidney diseases ^43, 44^, and recent studies in human airway smooth muscle cells have demonstrated that TGFβ1 induced autophagy is required for collagen and fibronectin production, while silencing of key autophagy-inducing proteins Atg5 and Atg7 leads to reduction in pro-fibrotic signaling and ECM protein release ^45, 46^. It is now believed that increased production of matrix proteins, as seen in airway remodeling, requires huge resources of energy, which is compensated by enhanced autophagy flux within the cell ^46^. Our own data (unpublished) demonstrates that there is a simultaneous induction of autophagy and pro-fibrotic signaling in human airway mouse models of allergic asthma. We propose that blockade of autophagy in the lungs can lead to reduction in airway fibrosis, as seen in airway remodeling in asthma and COPD. Recent observations suggest that autophagy exhibits a circadian rhythm and this rhythmic activation of autophagy is regulated by the transcription factor C/EBPβ ^47^. These findings need to be further evaluated in the context of asthma and COPD, as circadian rhythm genes can also be a potential target to modulate autophagy ^47^.

## Therapeutic strategies: Novel autophagy modulators

The mechanistic insight of disease pathogenesis in chronic airway diseases, such as asthma and COPD, is complex. Altered structural changes in the lung correlates with poor lung function, severity of disease and response to therapy in asthma and COPD. Current therapy does not target the underlying cause/s of the disease and does not restore the structural integrity of the airways ^12, 37– 41^; therefore, novel mechanisms, such as autophagy, pose attractive therapeutic options in airway remodeling. With the emerging role of autophagy in asthma and COPD and our increased understanding in the disease pathogenesis, we believe that the autophagy pathway can be exploited for therapeutic benefit. One of the exciting features of this pathway is that it can be inhibited at several steps, including initiation and vesicle elongation, autophagosome formation and maturation, and autophagosome lysosome fusion. Emerging evidence suggests that autophagy inhibition at the very first step is beneficial in an ovalbumin-induced model of asthma in mice, where 3-methyl adenine, a class III PI3K inhibitor, reduced the expression of autophagy marker LC3B with simultaneous reduction in airway eosinophilia ^19^. However, there is a need for more research in exploring the role of various PI3K inhibitors in the context of autophagy dependent-airway remodeling, using the most suitable models of asthma and COPD, as modulation of autophagy by the PI3K pathway can be an attractive target for both severe asthma and COPD ^48– 51^.

Bafilomycin A1 is a late phase potent inhibitor of autophagy, which acts through the inhibition of vacuolar type H ^+^-ATPase that is present on lysosomes. Inhibition of vacuolar type H ^+^-ATPase by bafilomycin A1 leads to acidification of lysosomes and inhibits their fusion with autophagosomes ^52^. Previous studies also indicate that bafilomycin inhibits the infection and airway inflammation induced by rhinovirus ^53^. Chloroquine is a well know anti-malarial and anti-rheumatoid agent, and it is also used for blocking autophagy at a late stage by lysosomal dysfunction (lysosomal lumen alkalizers) ^52^. The most important shortcoming of chloroquine is that this molecule blocks autophagy only at higher concentrations. Lys01 and Lys05 were recently developed modified versions of chloroquine, which are 10-fold more potent in autophagy inhibition ^52, 54, 55^; however these require further validation in asthma and COPD models.

Kinases, such as adenosine monophosphate activated protein kinase (AMPK) and ULK1 (mammalian orthologue of yeast protein kinase Atg1) are required for autophagy. AMPK senses nutrient deficiency and positively regulates ULK1, leading to activation of autophagy. Moreover, it has been reported that loss of either of these kinases results in defective autophagy (mitophagy) ^56^. Modulating the AMPK-ULK1 pathway may be an option for treatment of chronic airway diseases. Further recent observations suggest that autophagy exhibits a circadian rhythm and this rhythmic activation of autophagy is regulated by the transcription factor C/EBPβ ^47^. These findings need to be further evaluated in the context of asthma and COPD, as circadian rhythm genes can also be a potential target to modulate autophagy.

Thus, there is a need to identify the right autophagy mechanism in the progression and development of airway remodeling in asthma and COPD, which can become a realistic drug target to prevent structural changes in the lung.

## Conclusions and future perspectives

**Figure 2.**
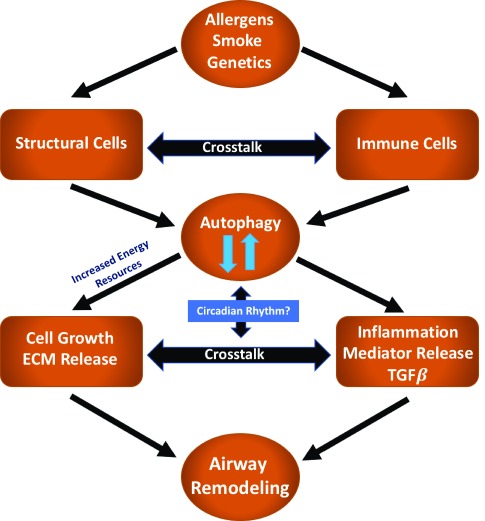
Autophagy mediates airway remodeling. An altered autophagy pathway is seen in response to cellular stress in asthma and chronic obstructive pulmonary disease (allergens or smoke or genetics), leading to activation and crosstalk between structural airway and immune cells. This further leads to impairment of autophagy causing degradation of intracellular constituents, providing an energy resource for ECM protein biosynthesis, and releasing mediators of inflammation and profibrotic signaling, which collectively lead to airway remodeling in the lung. Role of circadian rhythm genes in this context needs to be investigated. ECM: extracellular matrix; TGFβ: transforming growth factor-beta.

Based on the current scientific data, the autophagy pathway is directly linked to asthma and COPD pathophysiology and may act as a potential contributor to fibrotic airway remodeling, as described in Figure 2. Future research should be directed towards understanding the specific pathway and the mechanism/s leading to the pathophysiological activation of autophagy in disease. Efforts should be made in developing a modulator of autophagy as a new therapeutic strategy for treatment of airway remodeling in asthma and COPD. In the future, we propose to study the role of circadian rhythm genes in the activation of the autophagy pathway and understand whether modulating circadian rhythm genes, using novel small chemical compounds, has any therapeutic benefit in animal models of asthma and COPD.
