# Supplementary material for: Autophagy and airway fibrosis: Is there a link?
Source: F1000Res. 2018 Jun 11;6:409. Originally published 2017 Apr 3. [Version 2] doi: 10.12688/f1000research.11236.2 (PMC5416906; doi:10.12688/f1000research.11236.2)
Supplement: Peer review history

# Peer review: Referee response for version 2

**DOI:** 10.5256/f1000research.15658.r34897

**Published:** 2018-07-04

**Authors:** Eugene Roscioli

**Affiliations:** 1 Royal Adelaide Hospital - Hanson Institute, University of Adelaide, Adelaide, SA, Australia

As the initial reviewer, I am delighted with the level of corrections the author group has made to improve their review article. Further, I'd like to amend one statement and say a fine achievement for the Sharma group, in collaboration with Prof Oliver.

I have read this submission. I believe that I have an appropriate level of expertise to confirm that it is of an acceptable scientific standard.

# Peer review: Referee response for version 1

**DOI:** 10.5256/f1000research.12123.r21520

**Published:** 2017-05-04

**Authors:** Sukhwinder Singh Sohal

**Affiliations:** 1 Faculty of Health, School of Health Sciences, University of Tasmania, Launceston, Tasmania, Australia

**Summary **–

 The current manuscript has reviewed the literature on relationship between autophagy and airway fibrosis in chronic lung disease, especially asthma and COPD. This topic is of great interest and very little work has been done to understand the underlying mechanisms. I think this article is a timely reminder and will stimulate research into this area, which is much needed. Overall, this a well presented article but I have some suggestions, which will enhance readability and understanding on the diseases discussed.

**Major comments –**

1. Before authors go into the details for role of autophagy in asthma/remodeling, I think they need to add a separate section on airway remodeling and autophagy first.
2. Please list the airway remodeling changes described in asthma so far and then discuss which might be related to autophagy.
3. I think authors need to add a reference if there is any work done on fibroblast populations in asthma to make the case for fibrosis or suggest as potential future work.
4. Similar changes I think need to be made for the COPD section, especially listing airway remodeling changes described so far, for example Rbm fragmentation, vascularity,  epithelial mesenchymal transition (EMT) etc.
5. Authors stated in the evidence for autophagy in COPD section that major features are emphysema and chronic bronchitis. I think it is very important to describe small airway destructive pathology in COPD. The classic fixed airflow obstruction described in COPD comes from the small airways. These airways are destroyed by up to nearly 40% well before the diagnosis of COPD. So damage starts quite early on and it is this site where fibrosis really occurs and EMT is central to this. Airway disease is the primary phenomenon in COPD.
6. EGFR has also been regarded as one of the important drivers of airway remodeling in asthma and COPD, that needs to be discussed.

I have read this submission. I believe that I have an appropriate level of expertise to confirm that it is of an acceptable scientific standard.

# Response

**Published:** 2018-03-20

**Authors:** Pawan Sharma

**Affiliations:** UTS and the Woolcock Institute, Australia

**Reviewer 3: **We thank reviewer for constructive feedback. Reviewers’ comments are addressed one by one below (comments in italics followed by our response).

**C1:** *Before authors go into the details for role of autophagy in asthma/remodelling, I think they need to add a separate section on airway remodelling and autophagy first.*

**R1**: We have now added this to the text.

**C2 *:*** *Please list the airway remodelling changes described in asthma so far and then discuss which might be related to autophagy.*

**R2**: We have now added new text to the MS.

**C3 *:*** *I think authors need to add a reference if there is any work done on fibroblast populations in asthma to make the case for fibrosis or suggest as potential future work*.

**R3: **We have added references related to fibroblasts and potential future direction in this area.

**C4:** *Similar changes I think need to be made for the COPD section, especially listing airway remodelling changes described so far, for example Rbm fragmentation, vascularity, epithelial mesenchymal transition (EMT) etc.*

**R4:** We have now modified the text and added these suggestions as well.

**C5 *:*** *Authors stated in the evidence for autophagy in COPD section that major features are emphysema and chronic bronchitis. I think it is very important to describe small airway destructive pathology in COPD. The classic fixed airflow obstruction described in COPD comes from the small airways. These airways are destroyed by up to nearly 40% well before the diagnosis of COPD. So, damage starts quite early on and it is this site where fibrosis really occurs and EMT is central to this. Airway disease is the primary phenomenon in COPD.*

**R5: **These are valid points made by the reviewers, we have modified the text in the MS.

**C6 *:*** *EGFR has also been regarded as one of the important drivers of airway remodeling in asthma and COPD, that needs to be discussed.*

**R6: **We agree that EGFR is an important player in driving airway remodeling but the current review focusses on autophagy pathway and how it is linked to airway fibrosis.

# Peer review: Referee response for version 1

**DOI:** 10.5256/f1000research.12123.r22182

**Published:** 2017-05-02

**Authors:** Kimberley C.W. Wang

**Affiliations:** 1 Telethon Kids Institute, Subiaco, WA, Australia

Summary:

 This review clearly demonstrates the need for more studies investigating the role of autophagy in respiratory disease. It is good that the authors added some unpublished data into the review article as support of evidence.

 Major comments:

1. Figure 2 can be improved. Eg: at the arrow between Autophagy and Cell Growth/ECM release, the authors can add the words increase energy resource so the readers understand the association between Autophagy and Cell Growth/ECM release. Authors should consider adding ‘Genetics’ into the figure. The authors could add ‘circadian rhythm’ into the figure with a ‘?’ to show that it is a factor for further investigation.
2. For the collectively structural changes of airway remodeling, please include smooth muscle hyperplasia (James *et al*. 2012; Am J Respir Crit Care Med).
3. In the autophagy and fibrotic airway remodelling section, the paragraphs could be rearranged. Eg do not split TGFb1 between the paragraphs. The section about circadian rhythm seems to be a better fit in the Therapeutic strategies: Novel autophagy modulators section, as it is something to further evaluate as a potential target.
4. Are there any genetic component as evidence of autophagy in COPD? For example: Chen *et al.* 2015; J Formos Med Assoc.
5. Should have a reference for the sentence regarding TGFb1 leads to accumulation of ECM. ECM could be abbreviate earlier in the article.

I have read this submission. I believe that I have an appropriate level of expertise to confirm that it is of an acceptable scientific standard.

# Response

**Published:** 2018-03-20

**Authors:** Pawan Sharma

**Affiliations:** UTS and the Woolcock Institute, Australia

**Reviewer 2 **We thank reviewer for constructive feedback. Reviewers’ comments are addressed one by one below (comments in italics followed by our response).

**C1:** *Figure 2 can be improved. Eg: at the arrow between Autophagy and Cell Growth/ECM release, the authors can add the words increase energy resource, so the readers understand the association between Autophagy and Cell Growth/ECM release. Authors should consider adding ‘Genetics’ into the figure. The authors could add ‘circadian rhythm’ into the figure with a ‘?’ to show that it is a factor for further investigation.*

**R1: **We have modified the figure accordingly.

**C2:** *For the collectively structural changes of airway remodeling, please include smooth muscle hyperplasia (James et al. 2012; Am J Respir Crit Care Med).*

**R2: **We have now added this with the reference.     

**C3:** *In the autophagy and fibrotic airway remodelling section, the paragraphs could be rearranged. Eg do not split TGFb1 between the paragraphs. The section about circadian rhythm seems to be a better fit in the Therapeutic strategies: Novel autophagy modulators section, as it is something to further evaluate as a potential target.*

**R3: **It has been moved to the section on novel autophagy modulators.

**C4:** *Are there any genetic component as evidence of autophagy in COPD? For example: Chen et al. 2015; J Formos Med Assoc.*

**R4: **Not as far as we know.

**C5:** *Should have a reference for the sentence regarding TGFb1 leads to accumulation of ECM. ECM could be abbreviated earlier in the article*

**R5: **Reference added.

# Peer review: Referee response for version 1

**DOI:** 10.5256/f1000research.12123.r21469

**Published:** 2017-04-10

**Authors:** Eugene Roscioli

**Affiliations:** 1 Royal Adelaide Hospital - Hanson Institute, University of Adelaide, Adelaide, SA, Australia

*Summary statement*

 Kota *et al.* present an interesting and challenging subject for review – the interrelationship between autophagy in the context of airway diseases that exhibit airway remodelling as a major pathological feature. Challenging because we still have a lot to learn about both airway remodelling, and indeed the diseases such as asthma and COPD that potentiate fibrotic airway changes, and further still the contribution of increased or blocked autophagy as a driver, or parallel phenomena in this scenario. Overall they provide a needed and good start to the discussion, that will no doubt pioneer further debate in these areas off airway pathobiology. Hence, one leaves this review thinking about the link between fibrosis and autophagy, but also, wondering what insight they gained to direct new lines of inquiry.

*Major comments*

 As stated, this is by no means an easy literature review to compose. At first one would think this is due to limited publication of such studies. However, a PubMed search using “Fibrosis[Title] and Autophagy[Title]”, show there are 83 hits for this query. While the vast majority are not respiratory in nature, it would be beneficial if Kota *et al.* could use mechanistic insights from other diseases to draw hypothetical parallels with the situation that may be occurring in the airways, which they elude to in paragraph 2 under the heading “Autophagy and fibrotic airway remodelling”, but little supposition is made in regards to this thereafter. Indeed, this paragraph is possibly the only one which directly address the subject matter stated by the title. Refreshingly however, they generously state new unpublished finding (on three occasions) from their laboratory which provides insight for the reader and which have the potential to direct further lines if understanding.

 A current question for autophagy is: is it a block in autophagic flux leading to (or contributing to) the cellular dyshomeostasis, or is autophagy increased because it is unable to manage the protracted/chronic disease stressors? While Kota *et al.* touch upon this question for COPD, and correctly state that there is significant conjecture with regards to this, subsequent discussion mainly lent to the increased autophagy scenario, with an extended discussion with regards to cilia (ciliophagy). Indeed, we now know the relationship between cilia and autophagy is a complex dual interaction whereby they foster each other’s activity/generation rather than a one way situation where increased autophagy leads to the degradation of cilia (Pampliega *et al.*, 2016). Hence, some new insights/thoughts which could weigh into the autophagic flux vs. increased autophagy debate would be welcomed.

 Further, airway fibrosis occurs as a result of the chronic inflammation (in addition to e.g. altered smooth muscle-epithelia interactions, between themselves and the inflammatory cells), while autophagy is known to be an anti-inflammatory mechanism. Hence, beyond TGFβ, some insights into how the dysregulation of autophagy leads to fibrosis driven by complex inflammatory changes at the airways would have been informative.

 Hence, while this review most certainly warrants publication, and marks yet another achievement for the Oliver laboratory, the informed reader may ask for more than the start of the dialogue presented in this particular review.

*Minor comments*

 The manner of composition on occasions lacks maturity. For example, “ATG5 encodes a protein of 276 amino acids”, is not needed in a review article. The chronological presentation of concepts/studies, should be forfeited to a style of writing that groups and discusses processes to illustrate a pattern of phenomena that helps orient/inform the reader. Avoid using definite statements such as “clearly indicates”, particularly in a review, and especially in areas where we have so much more to learn such as is the case for chronic lung disease and autophagy. Finally, approximately one third of the 55 references are review articles.

I have read this submission. I believe that I have an appropriate level of expertise to confirm that it is of an acceptable scientific standard.

## References

[1] PampliegaOCuervoAM: Autophagy and primary cilia: dual interplay. *Curr Opin Cell Biol*.2016;39: 10.1016/j.ceb.2016.01.008 1-7 10.1016/j.ceb.2016.01.008 26826446PMC4733852

# Response

**Published:** 2018-03-20

**Authors:** Pawan Sharma

**Affiliations:** UTS and the Woolcock Institute, Australia

**Reviewer 1: **We thank reviewer for the constructive feedback. Reviewers’ comments are addressed one by one below (comments in italics followed by our response).

**C1:** *As stated, this is by no means an easy literature review to compose. At first one would think this is due to limited publication of such studies. However, a PubMed search using “Fibrosis[Title] and Autophagy[Title]”, show there are 83 hits for this query. While the vast majority are not respiratory in nature, it would be beneficial if Kota et al. could use mechanistic insights from other diseases to draw hypothetical parallels with the situation that may be occurring in the airways, which they elude to in paragraph 2 under the heading “Autophagy and fibrotic airway remodelling”, but little supposition is made in regard to this thereafter. Indeed, this paragraph is possibly the only one which directly address the subject matter stated by the title. Refreshingly however, they generously state new unpublished finding (on three occasions) from their laboratory which provides insight for the reader and which have the potential to direct further lines if understanding.*

**R1**: We agree with the reviewer about the complexity of the topic being reviewed. However, we would like to state that this mini-review is focussed on “lung-related” fibrotic changes in asthma and COPD. We haven’t discussed other lung diseases such because the autophagy pathway is quite distinct in those diseases which will make it difficult to bring them under the same category. This review has tried to discuss the dysregulated autophagy pathway in asthma and COPD pathology as literature suggests there are some parallels in autophagy signalling mechanisms. Further, TGFbeta driven fibrotic changes that are dependent and mediated through autophagy are discussed in this review which also occur in other organs such as liver and kidney fibrosis.

**C2** *. A current question for autophagy is: is it a block in autophagic flux leading to (or contributing to) the cellular dyshomeostasis, or is autophagy increased because it is unable to manage the protracted/chronic disease stressors? While Kota et al. touch upon this question for COPD, and correctly state that there is significant conjecture with regards to this, subsequent discussion mainly lent to the increased autophagy scenario, with an extended discussion with regards to cilia (ciliophagy). Indeed, we now know the relationship between cilia and autophagy is a complex dual interaction whereby they foster each other’s activity/generation rather than a one-way situation where increased autophagy leads to the degradation of cilia (Pampliega et al., 2016). Hence, some new insights/thoughts which could weigh into the autophagic flux vs. increased autophagy debate would be welcomed.*

**R2**. Reviewer has asked a very interesting question with relevance to autophagy: whether it is being blocked or increased during disease pathology. We believe it is too early to say that autophagy flux is blocked or there is a net increase in autophagy that could lead to disease pathology. Published literature in COPD suggests that that there is an increase in autophagy in disease, and this has been also demonstrated in the cigarette smoke-induced experimental COPD models with additional increase in ciliophagy contributing to reduced mucus clearance (Choi’s group). In asthma, it is not yet clear whether there is a block in autophagy flux or there is a net increase in autophagy, though recent data demonstrate increase number of autophagosomes in asthma that associates with lung function and ECM protein in humans (Poon et al). In experimental models of allergic asthma there is an evidence to believe that autophagy is increased in severe asthma models and inhibition of autophagy *in vivo* may be a viable option.

**C3**. Further, airway fibrosis occurs as a result of the chronic inflammation (in addition to e.g. altered smooth muscle-epithelia interactions, between themselves and the inflammatory cells), while autophagy is known to be an anti-inflammatory mechanism. Hence, beyond TGFβ, some insights into how the dysregulation of autophagy leads to fibrosis driven by complex inflammatory changes at the airways would have been informative.

**R3**. We agree with the reviewer that there are complex cell-cell interactions and multitude of signalling pathways which eventually contributes to structural changes in the airways. Keeping in mind the scope of this review we have focussed on TGFβ-dependent mechanisms that are related to the activation of autophagy in the lung. We have now added text to the MS to reflect these changes. 

**C4** *. The manner of composition on occasions lacks maturity. For example, “ATG5 encodes a protein of 276 amino acids”, is not needed in a review article. The chronological presentation of concepts/studies, should be forfeited to a style of writing that groups and discusses processes to illustrate a pattern of phenomena that helps orient/inform the reader. Avoid using definite statements such as “clearly indicates”, particularly in a review, and especially in areas where we have so much more to learn such as is the case for chronic lung disease and autophagy. Finally, approximately one third of the 55 references are review articles.*

**R4**: We have now modified the text in the MS to reflect these changes.
